# Circular RNAs in extracellular vesicles: Promising candidate biomarkers for schizophrenia

**DOI:** 10.3389/fgene.2022.997322

**Published:** 2023-01-06

**Authors:** Chuang Guo, Haibing Lv, Yulong Bai, Meng Guo, Pengfei Li, Shuping Tong, Kuanjun He

**Affiliations:** ^1^ College of Life Sciences and Food Engineering, Inner Mongolia Minzu University, Tongliao, China; ^2^ Network Center, Inner Mongolia Minzu University, Tongliao, China; ^3^ Affiliated Hospital of Inner Mongolia Minzu University, Tongliao, China

**Keywords:** schizophrenia, biomarkers, circular RNAs, extracellular vesicles, research progress

## Abstract

As one of common and severe mental illnesses, schizophrenia is difficult to be diagnosed exactly. Both its pathogenesis and the causes of its development are still uncertain because of its etiology complexity. At present, the diagnosis of schizophrenia is mainly based on the patient’s symptoms and signs, lacking reliable biomarkers that can be used for diagnosis. Circular RNAs in extracellular vesicles (EV circRNAs) can be used as promising candidate biomarkers for schizophrenia and other diseases, for they are not only high stability and disease specificity, but also are rich in contents and easy to be detected. The review is to focus on the research progress of the correlation between circRNAs and schizophrenia, and then to explores the possibility of EV circRNAs as new biomarkers for the schizophrenia diagnosis.

## Introduction

Schizophrenia is a severe mental disease which is caused by both environmental and genetics factors (Ershova et al., 2019). Schizophrenia affects about 1% of world’s population (Mueser and McGurk, 2004). More than 80%–90% of the inpatients in mental hospitals belong to schizophrenia patients (Mueser and McGurk, 2004). However, the *status quo* of schizophrenia diagnosis is that doctors primarily rely on their subjective perceptions and assessments of patients’ symptoms and signs. There are no biochemical or genetic biomarkers detected for the doctors to rely on. Therefore, to ascertain schizophrenia biomarkers constitutes a research hotspot in psychiatry. CircRNAs are endogenous and closed loop structure RNA molecules, which can regulate mammalian gene expression at different levels. CircRNAs are particularly abundant in mammalian brains, and participate in mammalian neurodevelopment and function, brain health maintenance, and prevention of neuropsychiatric diseases (Mahmoudi et al., 2019). It has been confirmed that abnormally expressed circRNAs are present in the different tissues of schizophrenia patients, and they may play important roles in the occurrence of schizophrenia (Mahmoudi et al., 2019). EVs are membranous vesicles that are actively secreted by cells, and are widely present in various body fluids. Since EV circRNAs have the characteristics of disease specificity, high stability, abundant contents, and easy to be detected, they can be used as potential biomarkers for many diseases including schizophrenia. Li, et al. (2020a) briefly reviewed circRNAs potential as diagnostic biomarkers for schizophrenia and depression (Li et al., 2020b). Singh, et al. (2022) carried out a review focusing on the circRNA expression and activities in different tissue samples (Singh et al., 2022). The present review is to provide a detailed study summary of circRNAs in schizophrenia and to explore the feasibility of EV circRNAs as new biomarkers for the diagnosis of schizophrenia.

### Circular RNAs

CircRNAs are a special class of endogenous circular single-stranded RNA molecules are formed in the process of splicing. Sanger et al. (1976) first discovered circRNAs in plants (Sanger et al., 1976) and they were initially considered to be typical by-products of mRNA post-transcriptional modification (Cocquerelle et al., 1993). With the evidence gathered, it has been found that circRNAs can not only be detected in different tissue samples and body fluids, but also are found to participate in the normal developmental processes, physiology, and disease states.

**FIGURE 1:**
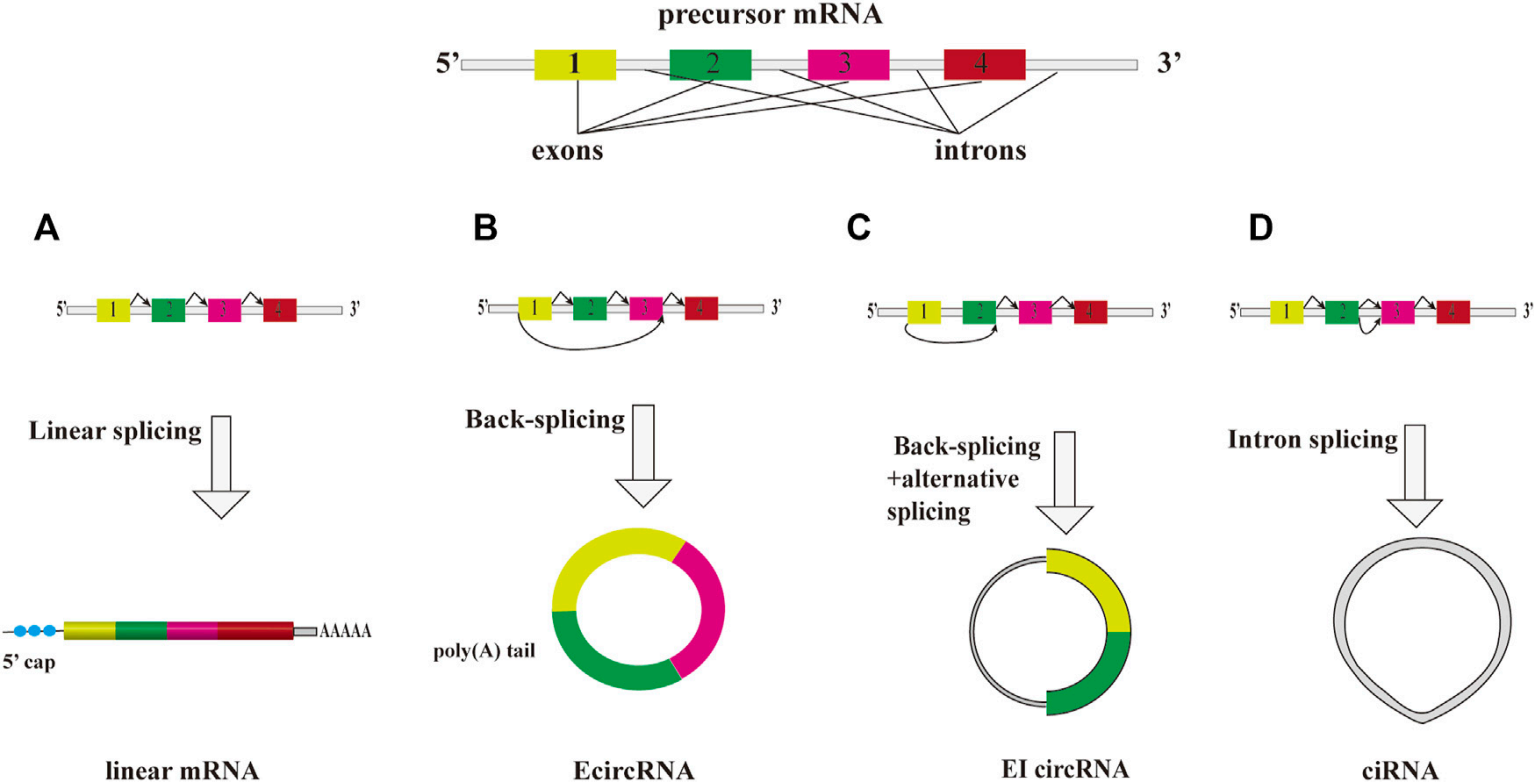
The overview diagram shows biosynthetic pathways of three major subclasses of circRNAs. A precursor mRNA (pre-mRNA) can produce a linear mRNA and circRNAs. **(A)** A precursor mRNA (pre-mRNA) can produce a linear mRNA undergo canonical splicing. Under back-splicing, it can produce a circRNA. They are three major subclasses of circRNAs, EcircRNA, ciRNA and EIcircRNA. **(B)** EcircRNA is the main group of circRNA class that consist of only exon(s). **(C)** cirRNA is derived from intron lariat. **(D)** EIcircRNA is composed of at least two exons and one retained intron.

Despite the fact that the expression levels of some exceptionally abundant circRNAs are higher than that of their cognate linear mRNAs (Salzman et al., 2013; Rybak-Wolf et al., 2015), the abundance of most of circRNAs are relatively lower than that of mRNAs in cytoplasm and circRNAs which exhibit diverse expression patterns in mammal tissues and cell types (Chen, 2020). A significant enrichment of circRNAs was observed in brains (Rybak-Wolf et al., 2015; Veno et al., 2015). Unlike linear RNAs, circRNAs contain covalently closed loops, which make them resistant to RNase R’s digestion. They are relatively stable molecules with a longer half-life and resistance to degradation than other RNA molecules (Enuka et al., 2016). According to their origin, they can be divided into exon circRNA (EcircRNA), intron circRNA (ciRNA) and exon-intron circRNA (EIcircRNA) (Figure 1). Therefore, they have potential to become the biomarkers and therapeutic targets for human diseases.

### Extracellular vesicles

Extracellular vesicles (EVs) are small diverse membrane vesicles limited by a lipid bilayer that are secreted by almost all kinds of cells into the extracellular space under both physiological and pathological states (Robbins and Morelli, 2014; van Niel et al., 2018; Gassama and Favereaux, 2021). The subtypes of EVs include exosomes, microvesicles, retrovirus like particles, and apoptotic bodies, etc. These EVs contain many kinds of factors including proteins, lipids and the genetic material from secreted cells to recipient cells. These factors are able to modify gene expression and activate immune responses of recipient cells at a distance (Robbins and Morelli, 2014; Gassama and Favereaux, 2021). EV is an additional mechanism for intercellular communication and can trigger multiple physiological and pathological processes such as immune surveillance and regulation of inflammation (Record et al., 2018).

The diameter of exosomes is about 30–150 nm that are actively secreted by cells to the outside and exist in most body fluids. Exosomes originate from the endosomal network. During endosomal maturation, intraluminal vesicles (ILVs) are generated by invagination of the limiting membrane of endosomes which result in the apparent selective sequestration of a small portion of cytosol (Stahl and Raposo, 2019). The endosomes that include ILVs are named multivesicular bodies (MVEs). MVBs can fuse with lysosomes for degradation of their contents or some multivesicular bodies fuse with the plasma membrane to release the ILVs into the extracellular environment. Once released, they are termed exosomes. Many different types of cells can secrete exosomes and it is a way of intercellular communication in normal and pathological states (Colombo et al., 2014). As a new mode of intercellular communication, exosomes can include biologically active macromolecules, transport from secreted cell to receptor cells, thereby finishing the exchange of genetic information between cells (Kalishwaralal et al., 2019). Many cells of the nervous system can release exosomes, such as neurons and glial cells can release exosomes (Von Bartheld and Altick, 2011; Chivet et al., 2013). Exosomes participate in the nervous system development and functions and the occurrence of neurological diseases (Osier et al., 2018; Zhang and Yang, 2018).

Microvesicles are another type of EVs which have different modes of the biogenesis of exosomes. Their formation occurs at the cellular plasma membrane during cell activation, apoptosis and mechanical injury (Deolindo et al., 2013). Microvesicles arise by outward budding and fission of plasma membrane that induced by translocation of phosphatidylserine to the outer-membrane leaflet (Akers et al., 2013; Raposo and Stoorvogel, 2013). Relative to exosomes, the size of microvesicles is large with the diameter range of 50–2,000 nm. Because they are also membranous vesicles, they can protect the contents from degradation and help them maintain biological activities in the process of long-term and long-distance transportation. By affecting autophagy, apoptosis and inflammation pathways, microvesicles and the cargos can change the biological effects of receptor cells (Lv et al., 2019). Neurons, astrocytes, microglia, and neural stem cells, have been described to release microvesicles and they involve in some central nervous diseases (Porro et al., 2015).

Apoptotic bodies are typically released from membrane blebs of apoptotic cells with the diameter ranging from 1,000 to 5,000 nm (Caruso and Poon, 2018). Apoptotic bodies are formed during the process of cell apoptosis process along with many intrinsic changes (Lu et al., 2005; Seo and Rhee, 2018). Apoptotic bodies are different from other subtypes of EVs and they encapsulate a spectrum of cargos, ranging from DNA fragments to intracellular components such as mitochondria (Xu et al., 2019; Kang et al., 2021). Retrovirus-like particles (RLPs) are non-infectious particles which resemble retroviral vesicles under Electron Microscope (EM). RLPs arise by direct budding from the plasma membrane (Bieda et al., 2001) and their diameter range are from 90 to 100 nm. A subset of retroviral proteins can be encapsulated in RLPs (Bronson et al., 1979; Mueller-Lantzsch et al., 1993; Dewannieux et al., 2005).

EVs are present in various biological fluids including peripheral blood, cerebrospinal fluid, urine, and saliva (Yuana et al., 2013). They can not only invade various tissues with the bloodstream, but also act as multimolecular messengers by an autocrine and paracrine manner. The uptake mode of EVs may be dependent on the type of cell and its physiologic state, and ligands on the surface of the EV and different cell types have different mechanisms of internalization (Abels and Breakefield, 2016).

It is well-known that intercellular communication is a vital event for multicellular organisms. Research about the intercellular communication mediated by EVs has attracted an increasing attention in recent years. EVs can play many roles in multiple biological processes. It is necessary to determine the potential physiological and pathological roles, clinical applications, and their relevance to disease. Because of their molecular cargos and easy accessibility, EVs have been suggested as enormous potential of biomarkers for the diagnosis of various diseases. EVs that released by cells of nervous system have been recognized as important modulators in the physiology of central nervous system and in neurodegenerative and neuroinflammatory disease states (Rufino-Ramos et al., 2017). At the same time, emerging evidences also indicates the potential utilities of EVs as early biomarkers for several brain disorders (Cheng et al., 2015; Zhao and Yang, 2021). EVs can pass the blood-brain barrier (Alvarez-Erviti et al., 2011). Recent studies suggested that brain-derived EVs were detected in rodent and human peripheral blood (Dickens et al., 2017; Mullins et al., 2017). Schizophrenia is generally considered to be a neurodevelopmental disorder of the brain. Increasing evidences suggested that inflammation contributes to occurrence and development of schizophrenia (van Beveren et al., 2014; Muller, 2018). EVs may contribute to the regulation of immune responses such as in autoimmune (Sáenz-Cuesta et al., 2014) and inflammatory diseases (Buzas et al., 2014) and infectious diseases (Silverman and Reiner, 2011). So, EVs are excellent candidate biomarkers for schizophrenia.

### Correlations between circRNAs and schizophrenia

CircRNAs play important role in neurodevelopment and function, maintaining brain health, and preventing neuropsychiatric diseases (Zhuo et al., 2020). Multiple studies have confirmed that circRNAs are important regulators in normal developmental processes, physiology, and disease states, including cancer, mental illness, and cardiovascular disease (Floris et al., 2017; Liu et al., 2017; Han et al., 2018). At the same time, some studies have also revealed the relationship between circRNAs and neurological diseases (Errichelli et al., 2017; Bai et al., 2018). It has been confirmed that circRNAs are involved in the occurrence and development of a variety of neuropsychiatric diseases, such as schizophrenia (Mahmoudi et al., 2019), depression (Cui et al., 2016), Alzheimer’s disease (AD) (Akhter, 2018; Huang et al., 2018), epilepsy (Gong et al., 2018; Li et al., 2018) and Parkinson’s disease (PD) (Kumar et al., 2018).

It has been confirmed that there are abnormally expressed circRNAs in the brains of schizophrenia patients, and dysregulation circRNAs are involved in the occurrence of schizophrenia (Table 1). Piwecka, et al. (2017) have demonstrated that CDR1as regulates microRNA levels in the mammalian brain and knocked out CDR1as could make abnormal neural activity and behavioral disorders in mice and show impaired prepulse inhibition (PPI) (Piwecka et al., 2017). PPI is an important behavioral parameter for the measurement of the sensorimotor gating function is perfect. Sensorimotor gating dysregulation has been found in patients with neuropsychiatric disorders. In addition, knockout of the CDR1as locus of modeled mice also affected mRNAs encoding proteins involved in the maintenance of the mouse sleep-wake cycle. The study provides indirect evidence for the possible involvement of CDR1as in the pathogenesis of schizophrenia (Piwecka et al., 2017). As a large class of post-transcriptional regulators, circRNAs have been shown to act as a miRNA sponge/inhibitor to reduce miRNA activities, they are so-called competing endogenous RNAs. CDR1as was verified to act as a powerful miR-7 sponge to reduce miR-7 activity in developing midbrain of embryonic zebrafish and mouse brain (Hansen et al., 2013; Memczak et al., 2013). Mahmoudi, et al. (2019) performed transcriptome sequencing of the dorsal lateral prefrontal cortex (DLPFC) of 17 schizophrenia patients and 18 healthy controls and found that the overall expression of circRNAs in the DLPFC of schizophrenia showed a downward trend, and verified that hsa_circ_HP1BP3-7, hsa_circ_PPP2CA-3, hsa_circ_LONP2-6, hsa_circ_TOP1-10, hsa_circ_VCAN-2, hsa_circ_GPR137B-3, hsa_circ_ZNF236-2, and hsa_circ_MYO9A-66 were significantly downregulated in post-mortem cortex of schizophrenia patients by qRT-PCR (Mahmoudi et al., 2019). The expression of circHomer1a was verified to downregulated in the prefrontal cortex (PFC) of schizophrenia patients and schizophrenia patient-derived neural cells (Zimmerman et al., 2020).

**TABLE 1:** The circRNAs abnormally expressed in different tissues of schizophrenic patients.

Author	Years	Main findings
Piwecka et al. (2017)	2017	The mice knocked out circRNA CDR1as could make abnormal neural activities and behavioral disorders, show a phenotype associated with neuropsychiatric disorders—impaired PPI, and also affected mRNAs encoding proteins involved in the maintenance of the mouse sleep-wake cycle. The study provides indirect evidence for the possible involvement of CDR1as in the pathogenesis of schizophrenia
Mahmoudi et al. (2019)	2019	The overall expression of circRNAs in the DLPFC brain region of schizophrenia showed a downward trend and verified hsa_circ_HP1BP3-7, hsa_circ_PPP2CA-3, hsa_circ_LONP2-6, hsa_circ_TOP1-10, hsa_circ_VCAN-2, hsa_circ_GPR137B-3, hsa_circ_ZNF236-2, and hsa_circ_MYO9A-66 were significantly down-regulated in post-mortem cortex of schizophrenia patients
Yao et al. (2019)	2019	The expression level of hsa_circRNA_101836, hsa_circRNA_102101, and hsa_ circRNA_104597 are significantly downregulated, whereas hsa_circRNA_103704 and hsa_circRNA_103102 are significant upregulated in schizophrenia patients compared with healthy controls. After 8-week antipsychotic treatment, the expression level of hsa_circRNA_104597 has changed to significant upregulation and indicated that hsa_circRNA_104597 can be schizophrenia diagnostic and therapeutic biomarker
Zimmerman et al. (2020)	2020	The circHomer1a was found to be declined considerably in the prefrontal cortex (PFC) of schizophrenia patients and schizophrenia patient-derived neural cells
Mahmoudi et al. (2021)	2021	22 circRNAs were verified to significant alteration in circulating PBMCs from individuals with schizophrenia
Tan et al. (2021)	2021	In plasma EVs of schizophrenia patients, 44 differentially expressed circRNAs were verified by high-throughput sequencing technology and the expression levels of chr3_196488683_196483770_-4913, hsa_circ_0077837, hsa_circ_0001495, hsa_circ_0074371 and hsa_circ_0042174 significantly down-regulated compared with six healthy controls by qRT-PCR.
Liao et al. (2022)	2022	In the peripheral blood of schizophrenia patients, 450 differentially expressed circRNAs were found by the whole transcriptome sequence technology and 5 circRNAs were confirmed by RT-qPCR.

At present, the study of schizophrenia-related peripheral blood circRNAs is still in its infancy. Abnormally expressed circRNAs were found in the peripheral blood of schizophrenic patients. Yao et al. (2019) verified that the expression level of hsa_circRNA_102101, hsa_circRNA_101836, and hsa_ circRNA_104597 are significantly downregulated, whereas those of hsa_circRNA_103102 and hsa_circRNA_103704 are significantly upregulated in peripheral blood mononuclear cells (PBMCs) of 102 schizophrenia patients compared with 103 healthy controls by RT-qPCR (Yao et al., 2019). After 8-week antipsychotic treatment, hsa_circRNA_104597 has changed to significant upregulation. So, the results indicated that hsa_circRNA_104597 can be schizophrenia diagnostic and therapeutic biomarker (Yao et al., 2019). Mahmoudi, et al. (2021) used deep RNA-seq technology to analyzed circRNA expression in PBMCs from 20 patients with schizophrenia, 19 patients with bipolar disorder as well as 20 controls. It showed that 22 and 33 circRNAs were significantly altered in PBMCs from individuals with schizophrenia and bipolar depression, respectively (Mahmoudi et al., 2021). Tan *et al.* (2021) found 44 differentially expressed circRNAs in the plasma exosomes of schizophrenia patients by high-throughput sequencing technology and verified that the expression levels of chr3_196488683_196483770_-4913, hsa_circ_0077837, hsa_circ_0001495, hsa_circ_0074371 and hsa_circ_0042174 were significantly downregulated in plasma EVs of six schizophrenia patients compared with six healthy controls by RT-qPCR (Tan et al., 2021). Liao, et al. (2022) used the whole transcriptome sequence technology to assess the expression profiles of circRNAs in the peripheral blood of three schizophrenia patients and three healthy controls, they found that 450 differentially expressed circRNAs were found to aberrantly express in the peripheral blood of schizophrenia patients (Liao et al., 2022). They further confirmed five circRNAs aberrantly expressed by RT-qPCR (Liao et al., 2022).

The lack of commonality was found in previous studies. The reasons for discordant results between previous studies are as follows. Firstly, the samples that used in these researches are different, some being peripheral blood, others PBMCs and still others plasma EVs. Although Yao *et al.* (2019) (Yao et al., 2019) and Mahmoudi et al. (2021) (Mahmoudi et al., 2021) used the same samples, the methods of them were different. Secondly, the sample numbers are small in Tan et al. (2021) and Liao et al. (2022).

Although the above studies have confirmed that circRNAs are significantly associated with schizophrenia, the roles in the pathogenesis of schizophrenia that circRNAs play are still a mystery and needs to be studied systematically. In addition, the associations of differentially expressed circRNAs with schizophrenia were mostly confirmed in brain. Since it is difficult to obtain samples from living human brain, biomarkers that abnormally expressed in brain tissue are difficult to be used for early warning and diagnosis of schizophrenia. They prompted scientists to verify biomarkers of schizophrenia from peripheral blood, especially from the EVs of peripheral blood. However, whether circRNAs in peripheral blood can accurately reflect the states of the brain and whether they are as specific biomarkers for brain-related diseases need further examination.

### EV circRNAs as schizophrenia biomarkers

**FIGURE 2:**
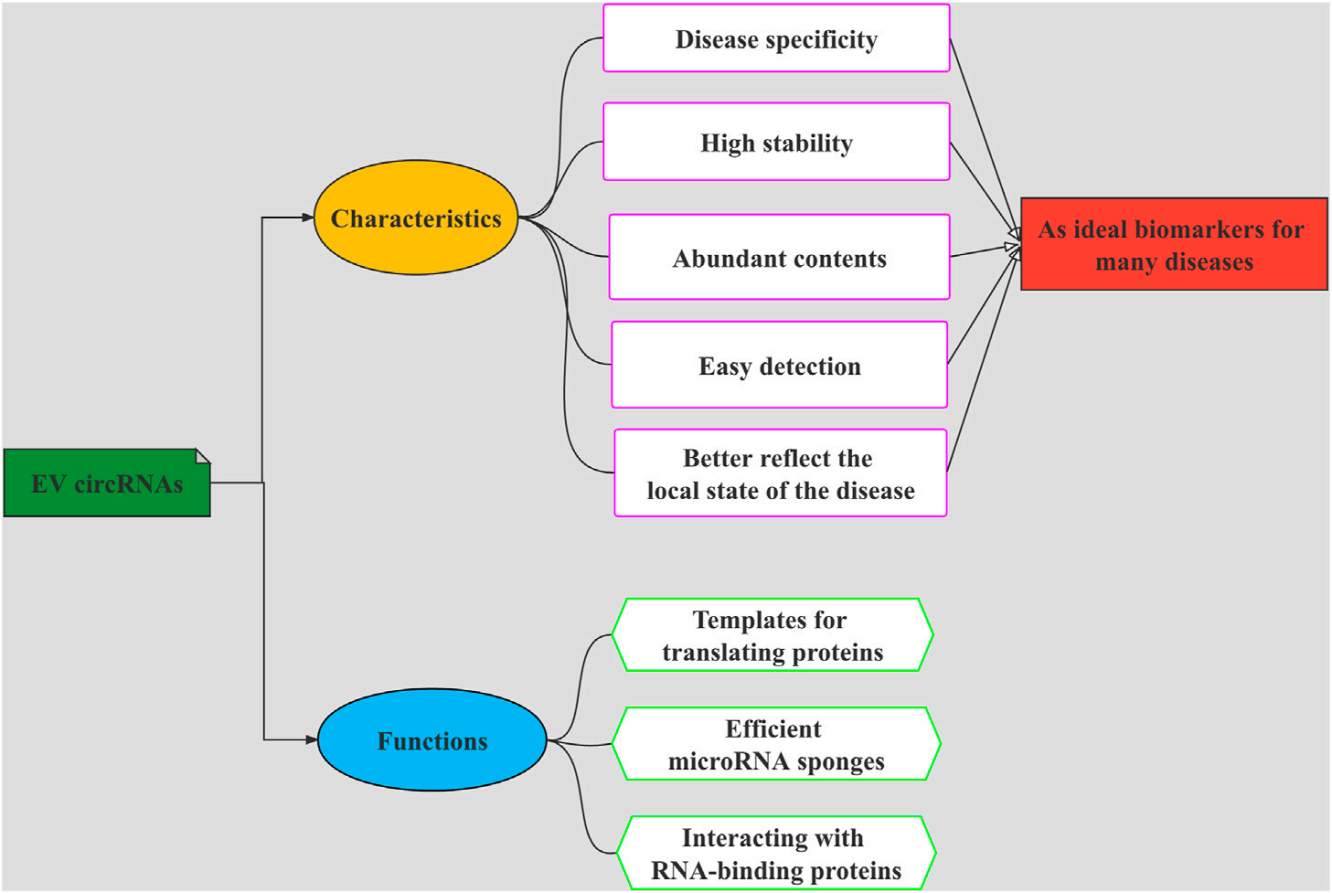
The overview diagram shows multiple characteristics and biological functions of EVs-circRNAs. The functions of EV circRNAs are as templates for translating proteins, efficient microRNA sponges, and interacting with RNA-binding proteins. EV circRNAs have such characteristics, as disease specificity, high stability, abundant contents, and easy to be detected. These functions and characteristics make them to be used as promising candidate biomarkers for many diseases.

Biomarkers are biochemical and physiological indicators that have a very wide range of uses including change marking in the structure or function of cells, tissues, organs, and systems. The contents of EVs mirror the aspects of the secreting cell, including genetic and proteomic aspects (Akers et al., 2013). As a new mode of intercellular communication, EVs carry diverse molecules such as DNA, RNAs, protein, lipids, metabolites, and others, which transport from host cells to recipient cells, thereby affecting the function of recipient cells (van Niel et al., 2018; Kalishwaralal et al., 2019; Mathieu et al., 2019). In recent years, studies have shown that EVs are important carriers of circRNAs. The abnormally expressed circRNAs in the brain of schizophrenia patients can be transported through EVs, cross the blood-brain barrier, enter the circulatory system and exist stably (Zhuo et al., 2020). Due to the protective effect of EV membrane, the existence of circRNAs is more stable and abundant, and can better reflect the local state of the disease. RNA sequencing revealed that circRNAs are abundant in EVs of human Blood (Li et al., 2019). EV circRNAs have such characteristics, as disease specificity, high stability, content abundancy, and easy detection; these features make them to be used as promising candidate biomarkers for many diseases and have great potential and important significance in the development of new disease diagnosis and treatment methods. The overview diagram showing multiple characteristics and biological functions of EV circRNAs can be seen in Figure 2. Existing studies have linked EV circRNAs with tumors, confirming that they are excellent biomarkers for tumor diagnosis (Pan et al., 2019; Li et al., 2020a).

Most of the research on EV circRNAs focusing on tumors, and their correlations with psychiatric diseases is still in its infancy. Although the fact that the expression of EV circRNAs synchronizing with that of the brain and can accurately reflecting the abnormal pathological state of the brain need to further verify, we must admit that EV circRNAs are promising candidates as biomarkers for neuropsychiatric diseases, especially for schizophrenia. However, the research on EV circRNAs as the biomarkers of psychiatric and neurological disorders is in its infancy, only a small number of literatures paying attention to EV circRNAs as biomarkers of schizophrenia. Only the study of Tan et al. (2021) explored plasma exosomal circRNAs as diagnostic biomarkers for schizophrenia and found that some circRNAs have the potential as diagnostic biomarkers and the therapeutic strategy for schizophrenia (Tan et al., 2021).

EV circRNAs participate in the occurrence and development of schizophrenia, so they have important scientific and clinical application values. Further and in-depth research on them will not only be conducive to the diagnosis, treatment and prognosis of schizophrenia, but also help to reveal the pathogenic mechanism of schizophrenia. With the further discoveries of more and more psychiatric disease-related EV circRNAs and the identification of their regulatory mechanisms, the role of EV circRNAs in the pathological mechanisms of schizophrenia can be clarified. Therefore, it is important to carry out the research of investigating the expression profile of circRNAs in EVs of patients with schizophrenia, exploring the feasibility of differentially expressed EV circRNAs as diagnostic markers for schizophrenia, and verifying biological pathways related to the occurrence of schizophrenia that EV circRNAs participate. Despite the fact that biology and utility of EVs have attracted much attention, the researches of EVs is still in the initial stage. Further research on EV circRNAs in schizophrenia may help to understand intercellular communications in diseased brains, and to find novel biomarkers and new therapeutic strategies in schizophrenia.

## Conclusion

EV circRNAs participate in the pathogenesis of schizophrenia, have important scientific and clinical application values. Exploring EV circRNAs involvement in the occurrence and development of schizophrenia has increasingly become the direction of the diagnosis and treatment of schizophrenia. With the continuous improvement of detection, evaluation and intervention technologies, the relation studies between EV circRNAs and schizophrenia will not only help the diagnosis and treatment of schizophrenia, but also provide reference for the diagnosis and treatment of other psychiatric diseases. It is expected to become a research hotspot of mental illness.

## Abbreviations

DLPFC, Dorsal Lateral Prefrontal Cortex; PFC, Prefrontal Cortex; PBMCs, Peripheral Blood Mononuclear Cells (PBMCs); EM, ectron Microscope; EVs, Extracellular vesicles; ILVs, Intraluminal Vesicles; MVEs, multivesicular bodies; RLPs, Retrovirus-like particles.
